# Effects of 4 Weeks of a Technique-Specific Protocol with High-Intensity Intervals on General and Specific Physical Fitness in Taekwondo Athletes: An Inter-Individual Analysis

**DOI:** 10.3390/ijerph18073643

**Published:** 2021-03-31

**Authors:** Alex Ojeda-Aravena, Tomás Herrera-Valenzuela, Pablo Valdés-Badilla, Jorge Cancino-López, José Zapata-Bastias, José Manuel García-García

**Affiliations:** 1 Department of Physical Activity Sciences, Universidad de los Lagos (ULA), 5290000 Osorno, Chile; 2 Faculty of Sports Sciences, Universidad de Castilla-La Mancha (UCLM), 45071 Toledo, Spain; JoseManuel.Garcia@uclm.es; 3 School of Sport Sciences, Universidad Santo Tomás (UST), 8370003 Santiago, Chile; tomas.herrera@usach.cl; 4 School of Physical Activity, Sport and Health Sciences, Universidad de Santiago de Chile (USACH), 8370003 Santiago, Chile; 5 Department of Physical Activity Sciences, Faculty of Education Sciences, Universidad Católica del Maule, 3530000 Talca, Chile; pvaldes@ucm.cl; 6 Exercise Science Laboratory, School of Kinesiology, Faculty of Medicine, Universidad Finis Terrae, 8370003 Santiago, Chile; jcancino@uft.cl; 7 Sports Coach Career, School of Education, Universidad Viña del Mar, 2520000 Viña del Mar, Chile; jzapata@uvm.cl

**Keywords:** martial arts, athletes, physical fitness, body composition

## Abstract

The aim of this research was to compare the effects of a technique-specific high-intensity interval training (HIIT) protocol vs. traditional taekwondo training on physical fitness and body composition in taekwondo athletes, as well as to analyse the inter-individual response. Utilising a parallel controlled design, sixteen male and female athletes (five females and 11 males) were randomly divided into an experimental group (EG) that participated in the technique-specific HIIT and a control group (CG) that participated in traditional taekwondo training. Both groups trained three days/week for four weeks. Squat jump (SJ), countermovement jump (CMJ), 5-metre sprint (5M), 20-metre shuttle run (20MSR), taekwondo specific agility test (TSAT), multiple frequency speed of kick test (FSKT_MULT_), total kicks, and kick decrement index (KDI), as well as body composition were evaluated. Results indicate that there are no significant differences (*p* > 0.05) in the factors group and time factor and group by time interaction (*p* > 0.05). Although percentage and effect size increases were documented for post-intervention fitness components in TSAT, total kicks, KDI, and 20MSR, responders and non-responders were also documented. In conclusion, a HIIT protocol based on taekwondo-specific technical movements does not report significant differences in fitness and body composition compared to traditional taekwondo training, nor inter-individual differences between athletes.

## 1. Introduction

Olympic taekwondo is described as a modern and constantly evolving combat sport whose performance requires athletes to develop and maintain a high level of physical fitness as part of their preparation [1]. Therefore, it is important to understand the characteristics of the components involved in physical performance in this sport in order to apply appropriate training stimuli in the preparation of athletes. In this sense, taekwondo, specifically the combat modality, is classified as an activity of an intermittent nature (effort: pause ratio: 1:7 to 1:2) [2] of high physiological intensity (>90% HRmax: lactate 5.0 to 14 Mmol L^−1^) with motor actions executed at high speed, mainly of the lower limbs [2,3]. In turn, in metabolic terms, it is described as a mixed sport, in which athletes use different proportions of energy substrates during combat (aerobic component: 58–66%, ATP-PCr: 26–30%, and glycolytic 4–5%) [4]. In addition to the above, competition categories are divided by body weight, which means that body composition has an important role [4,5].

Accordingly, considering the specific characteristics of this sport, the application of the high-intensity interval training (HIIT) model in the field of athletic performance in team and individual sports has grown exponentially in recent years [6]. Evidence in combat sports includes systematic reviews with [7] and without [8] meta-analyses, showing significant increases (*p* < 0.05) in cardiorespiratory capacity, physiological parameters, and physical skills, without reporting conclusive data on body composition. Specifically, in taekwondo, HIIT studies include the application of protocols based on repeated sprints [9,10] and recently HIIT protocols with specific technical characteristics [11,12]. However, these reports use interventions that add sessions to the usual training, which could influence the results of the studies analysed. On the other hand, only a few authors [11,12]. have documented increases in general and specific physical fitness using the temporal structure of combat as a work interval in the application of HIIT protocols [13]; however, the results are still controversial. Another element to consider is that usually the results of sport science interventions are interpreted in group terms, without considering the inter-individual variability of the athletes’ response [14]. To address this limitation, some authors have dealt with this situation by classifying athletes into two types: responders (Rs) and non-responders (NRs) [14,15]. For example, Bonafiglia et al. [16] analysed the inter-individual response comparing sprint interval training with endurance training for three weeks using a crossover design on physiological parameters in recreationally physically active adults, reporting individual increases in both training protocols. In addition, Ramirez-Campillo, R et al. [14] analysed the inter-individual response after seven weeks of plyometric training on components of athletic performance in football players, documenting greater individual increases in physical fitness parameters with respect to habitual training. In contrast, to the best of our knowledge, studies in combat sports are unknown.

From the perspective of optimising training processes, HIIT shock microcycles show a positive impact on athletic development [17]. In this context, incorporating technique-specific HIIT protocols into regular training sessions could be effective in optimising physical preparation time, bearing in mind the multiple annual competitions taekwondo athletes face. Moreover, it represents a training modality similar to the intermittent and physiological characteristics of this sport, which may be useful for training dosage and control.

Consequently, the main purpose of the present study was to compare the effects of a technique-specific HIIT protocol vs. traditional taekwondo training on general and specific physical fitness and body composition in taekwondo athletes and specifically, to analyse the inter-individual response of the athletes. The hypothesis of this study is that the technique-specific HIIT protocol would be significantly superior to traditional training for general and specific physical fitness components and modify body composition in taekwondo athletes. In addition, a higher rate of responders would be expected in the technique-specific HIIT group than in the traditional training group.

## 2. Material and Methods

### 2.1. Experimental Approach to the Problem

**Figure 1:**
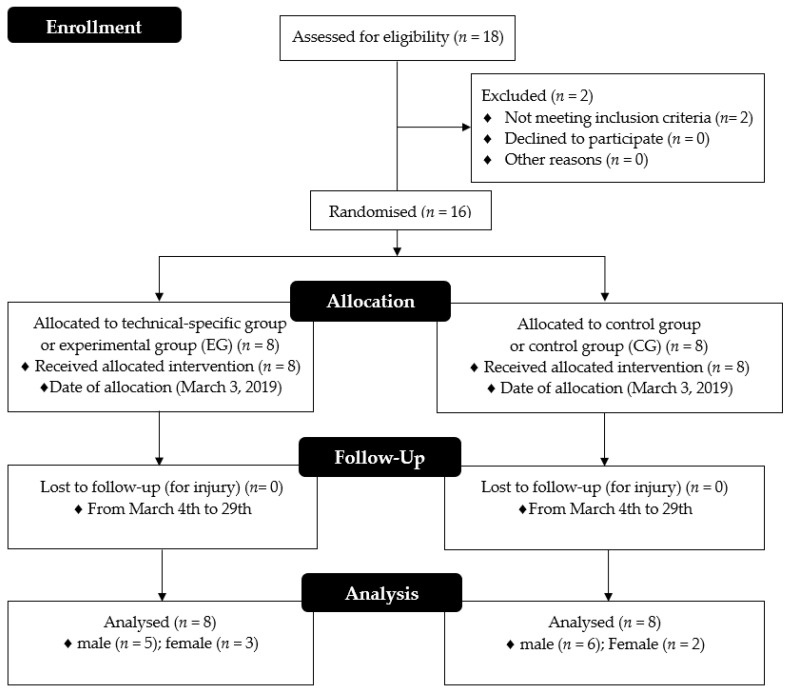
Participants flow.

The present study used a parallel controlled, randomised, single-blind, non-probabilistic convenience sample design. Together with the coach, a HIIT protocol was designed and planned that adhered to the usual training session, with specific technical characteristics based on repeated kicks, using the taekwondo temporal structure (effort: pause 4 s: 28 s; 1:7) of the HIIT work interval and a workload similar to the total duration of the combat (10 min). The athletes were distributed in experimental group (EG; *n* = 8) and control group (CG; *n* = 8) (for details see Figure 1). The effect of this training on components of the general and specific physical fitness and body composition in taekwondo athletes of both sexes was compared with the effect of a traditional taekwondo training. Athletes were invited to participate in the study before the end of the pre-season general physical preparation period (March 2019). The increase in the external training load during the application of the HIIT programme was based on the decrease in the pause times without modifying the work time. In turn, the internal training load considered the intensity, which was controlled by means of the rate perceived exertion or RPE (RPE 0–10) in both study groups. The HIIT group used the all-out format for the application of the training protocol [18].

### 2.2. Participants

Sixteen taekwondo athletes of both sexes voluntarily completed this study: five females with a mean age of 17.4 ± 2.9 years, height of 158.6 ± 6.9 cm, body mass of 58.6 ± 9.0 kg, and experience of 5.8 ± 1.7 years; and 11 males with a mean age of 20.5 ± 5.0 years, height of 169.5 ± 11.4 cm, body mass of 61.6 ± 12.7 kg, and experience of 7 ± 2 years, who compete annually in national-level tournaments. All athletes had to meet the following inclusion criteria to participate in the study: (i) four or more years of experience competing in taekwondo; (ii) training three or more times per week; (iii) preparing for competitions or tournaments organised by the Federación Nacional de Taekwondo Deportivo (FEDENAT, Chile), an organisation recognised by World Taekwondo; (iv) being enrolled in a club affiliated to FEDENAT; (v) all taekwondo athletes had to be free of injuries and neuromuscular problems in the last ten weeks; (vi) not being in a period of body mass reduction. All athletes and/or family members of athletes under 18 years of age were informed in advance of the study objectives, associated benefits, experimental procedures, and potential risks and provide informed consent or informed assent prior to the assessments and training sessions. The study was conducted in accordance with the Declaration of Helsinki on work with humans [19] and implemented after approval by the institutional ethics committee.

### 2.3. Assessments

#### 2.3.1. Jump Ability

Jump ability was assessed by the squat jump (SJ) and countermovement jump (CMJ) tests through the maximum height reached (cm) using an electronic contact platform (Ergojump; Globus, Codogne, Italy; accuracy: 0.01 m). The SJ test was used to evaluate dynamic muscle-shortening actions [20]. For this, each athlete was previously instructed to place their hands on their hips, feet, and shoulders wide apart, and adopt a bent-knee position (approximately 90°) for 3 s, and then perform a maximal effort vertical jump. Meanwhile, the CMJ test was used to assess dynamic muscle actions, specifically the slow stretch-shortening cycle [20]. Prior to this, each athlete was instructed to rest hands on hips, feet and shoulders wide apart, and perform a downward movement (no restriction was placed on the knee angle achieved) followed by a maximal effort vertical jump [21]. Two trials were completed, with a one-min pause between attempts [22], and the highest performing trial was used for subsequent statistical analysis (intra-class correlation; ICC SJ pre = 0.90 [CI95% 0.76 to 0.96]; ICC SJ post = 0.92 [CI95% 0.80 to 0.97]), (ICC CMJ pre = 0.95 [CI95% 0.90 to 0.98]; ICC CMJ post = 0.95 [CI95% 0.90 to 0.98]).

#### 2.3.2. Linear Sprint in 5 Metre (5M)

The speed to complete a linear sprint from 0 to 5 m was recorded using electrical photocells (Brower Timing System, Salt Lake City, UT, USA; accuracy: 0.001 s). Photocells were positioned 0.5 m after the starting line and 0.7 m above the floor (i.e., at hip level) to capture trunk movement rather than a false trigger of a limb [22]. Each participant placed the front foot 0.5 m before the first timing gate and began running when ready, thus eliminating reaction time [22]. Athletes performed two practice trials run at submaximal intensity after a thorough warm-up to familiarise them with the test. Three min after the warm-up, they completed two trials with two min of passive pause between trials, using the highest performing trial of 5M for subsequent statistical analysis (ICC pre = 0.85 [CI95% 0.58 to 0.93], ICC post = 0.87 [CI95% 0.62 to 0.94]).

#### 2.3.3. Taekwondo Specific Agility Test (TSAT)

Specific agility was assessed through the taekwondo-specific agility test (TSAT) following previous recommendations [23]. From a guard position with both feet behind the start/finish line, the performer had to (a) move forward in guard position, without crossing feet, as quick as possible to the centre point; (b) turn toward partner 1 by adopting a lateral shift and perform a roundhouse kick with the left leg (i.e., leading-roundhouse kick; *dollyo tchagui*); (c) move toward partner 2 and perform a roundhouse kick with the right leg (i.e., leading-roundhouse kick; *dollyo-chagi*); (d) return to the centre; (e) move forward in guard position and perform a double-roundhouse kick (i.e., *narae-chagi*) toward partner 3; and (f) move backward to the start/finish line in a guard position. Sparring partners 1 and 2 hold a kick-target, whereas partner 3 holds two kick-targets. Sparring partners were instructed to maintain the kick-target at the torso height of the tested athlete. If a participant failed to follow these instructions (e.g., crossed one foot in front of the other during the various displacements, or failed to touch the kick-target powerfully when kicking), the trial was terminated and restarted after a three-minute recovery period. The time needed to complete the test was used as performance outcome, and it was assessed with an electronic timing system (Brower Timing Systems, Salt Lake City, UT, USA). Two trials were accorded to each athlete, with the best one maintained for later analysis (ICC pre = 0.90 [CI95% 0.87 a 0.92], ICC post = 0.86 [CI95% 0.78 a 0.87).

#### 2.3.4. Multiple Frequency Speed of Kick Test (FSKT_MULT_)

The ability to repeat specific high-intensity efforts was assessed using the FSKT_MULT_ test designed for taekwondo following previously described protocols [24]. Each of the five FSKT_MULT_ sets had a duration of 10 s, with a pause interval of 10 s between sets. To perform the FSKT_MULT_, each athlete faced a partner using a trunk protector (breastplate). After the sound signal, the athlete performed the maximum number of kicks possible, alternating right and left legs. Performance was determined by the number of kicks in each series, the total number of kicks (total kicks), and the kick decrement index (KDI) during the test. The KDI indicates that the performance decreases during the test. To calculate the KDI, the number of kicks applied during the FSKT_MULT_ was taken into account. The calculation was performed using an equation that considers the results of all frequency speed of kick test (FSKT) series (Equation (1)).

(1) $$\mathrm{KDI}\;\left(\%\right)\;=\left[1-\;\frac{\mathrm{FSKT}1\;+\;\mathrm{FSKT}2\;+\;\mathrm{FSKT}3\;+\;\mathrm{FSKT}4\;+\;\mathrm{FSKT}5}{\mathrm{Best}\;\mathrm{FSKT}\;\times \;\mathrm{Number}\;\mathrm{of}\;\mathrm{Sets}}\right]\;\times \;100$$

#### 2.3.5. 20-Metre Shuttle Run Test (20MSR)

Aerobic fitness was assessed indirectly through the 20-metre shuttle run (20MSR) test according to the procedures of Leger and Lambert [25] and previous studies in taekwondo [12]. For its execution, athletes had to run back and forth between two lines separated by 20 m, at a pace set by an audio signal from an electronic recording. Each run was successful upon completion of the 20 m distance. The signal sounded at an increasing pace with each minute of the test, at which point the athletes had to increase their speed. They were warned once when they failed to reach the finish line within a certain period of time. The test was terminated when the examinee (i) could not follow the set pace of the signal for two successive runs; or (ii) when he/she voluntarily stopped. Scores were expressed as the last minute that athletes managed to complete during the test. One trial was completed, which was used for subsequent statistical analysis.

#### 2.3.6. Anthropometric and Body Composition Assessments

Height (cm) was assessed with a stadiometer (Bodymeter 206, SECA, Germany, accuracy 1 mm) following standard protocols [26]. Each athlete stood without shoes, with heels together, back and buttocks touching the vertical surface of the stadiometer and head positioned in the Frankfort plane. Body composition, including body mass (BM), percentage fat mass (FM%), fat mass (FM), and muscle mass (MM), was assessed using an electrical bioimpedance scale (InBody120, 20 100 kHz tetrapolar tactile electrode sys-tem, model BPM040S12F07, Biospace, Inc, Seoul, Korea with an accuracy of 0.1 kg, with a measurement range of 5 to 250 kg and suitable for individuals aged 3 to 99 years) [27,28]. InBody technology divides the body into five components: two arms, two legs, and a trunk. Electrodes are placed under the subject’s feet on the platform and on the palms and thumbs attached to the handles of the device. Age, height, and gender are entered manually after weight is determined using a scale placed inside the device. Body mass and impedance are automatically assessed using the manufacturer’s software. Equations provided in the manufacturer’s proprietary software calculated body composition characteristics [29]. Briefly, athletes stood barefoot and lightly clothed on the base components of a bioimpedance analyser with both feet and both thumbs placed on the electrodes and arms held away from the body at approximately 15° [30]. Once proper positioning of the device was achieved, the athlete was asked to remain still and quiet while the device completed the body composition measurement, which took an average of 30 s to one min. The researchers administered and monitored the entire test to ensure that the athlete maintained proper positioning and did not move [31,32].

#### 2.3.7. Training Programme

Both groups participated in a 12-session (4-week) training programme with a duration of 90 min per session, which took place on three non-consecutive days (Monday, Wednesday, and Friday) and took into consideration a distribution of the training load, with an emphasis on technical–tactical development. Previously, both groups were instructed to use the scale of perceived exertion (RPE 0–10) to control the internal load during the application of the work protocols. Each training session started with 10 min of gentle jogging in a circle, joint mobility, and dynamic flexibility. Subsequently, for 20 min in pairs, all athletes performed a technical work sequence of front, spin, and circular kicks using a speed paddle. Then, for 30 min, all athletes performed adapted fights with technical specifications (i.e., task assignment), during which the coach intervened whenever necessary, giving tactical indications related to guarding, space distribution, technical gestures, and offensive and defensive situations. After 60 min, the experimental group (EG) was removed from the group of athletes to execute a technical-specific HIIT protocol with a volume of ≈10 min. Specifically, the EG performed a HIIT programme with 4 s of effort followed by 28 s of pause (effort: pause ratio 1:7) using alternating roundhouse kicks with both legs at maximum intensity (i.e., all-out) considering an RPE of 10 in front of a partner. This was followed by periods of active recovery mimicking the guard stance, which were distributed in three rounds of two min of activity for one minute of passive rest between rounds. During the passive rest, they hydrated and simulated receiving instructions from the coach and assistants. Meanwhile, the control group (CG) performed 10 min of technical kicking work using speed paddles and simulated sparring at moderate intensity (RPE 5–6). Finally, both groups concluded the sessions with a cool down consisting of static stretching exercises for 20 min. The total duration of all training sessions was one and a half hours (for details, see Table 1).

### 2.4. Procedures

**Figure 2:**
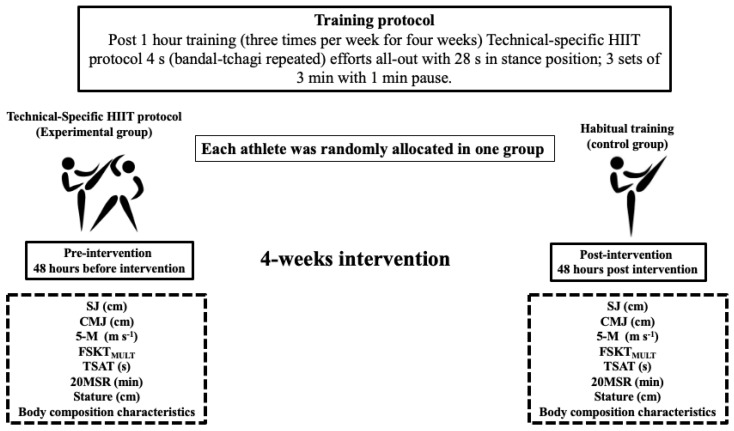
Training protocol. SJ: squat jump; CMJ: countermovement jump; 5M: linear sprint in 5 m; FSKT_MULT_: multiple frequency speed of kick test; TSAT: taekwondo specific agility test; 20MSR: 20-metre shuttle run.

The CG was composed of eight taekwondo athletes, distributed in six males and two females. The EG was composed of eight taekwondo athletes, distributed in five males and three females. For randomisation, a block randomisation with a block size of four athletes was applied. Eligible athletes were randomly assigned, after completion of baseline assessments, to the control group or to HIIT training. The principal investigator coordinated the allocation sequence, and randomisation of the two study arms was electronically generated [33]. All athletes and study personal (including investigators and coach) were blinded to treatment allocation throughout the trial protocol. During the previous week, athletes completed a familiarisation session with the HIIT protocol and assessments to reduce the learning effect. Assessments were performed before and after the application of the training programme, with 48 h of rest between the first and the last training session. All assessments were scheduled between 9:00 h and 11:00 h, completed in the same order, in the same venue (gymnasium with wooden floor), with the same sports clothing and by the same pre- and post-intervention assessor, who was a qualified sports scientist blinded to the intervention group and assigned to the athletes. Previously, all athletes were instructed to (i) sleep for 7 to 8 h before each assessment session and (ii) not to modify their usual diet and hydration habits during the days prior to the assessments. The first session assessed chronological age, bipedal height, and body composition in the fasting state. The second assessment session considered the components of general and specific physical fitness, using the following assessments: squat jump (SJ), countermovement jump (CMJ), taekwondo specific agility test (TSAT), 5-metre linear sprint (5M), multiple frequency speed of kick test (FSKT_MULT_), and 20-metre shuttle run (20MSR) (see details in Figure 2). A typical warm-up in this sport was performed, of 15 min duration, consisting of joint mobility, gentle jogging for five min, dynamic stretching (three min), three SJ and CMJ trials (two min), and low-intensity kicking (five min). Athletes were previously instructed to give their maximum effort during the assessments. The best of two attempts was considered for performance on all assessments, except for the FSKT_MULT_ and 20MSR tests. A two-min pause interval between attempts was implemented and a rest interval of five to 10 min was applied between each assessment to reduce fatigue effects.

### 2.5. Statistical Analysis

Data analysis was performed with SPSS software version 24 (SPSS Institute, Chicago, IL, USA). All data are presented as mean ± standard deviation. Homoscedasticity of variance was verified using Levene’s test. To quantify the reliability of SJ, CMJ, and 5M, an ICC with a 95% confidence interval (95%CI) was used. An acceptable reliability was determined with an ICC of 0.80 [34]. To determine possible differences in the characteristics on physical fitness and body composition components between the groups, an unpaired *t*-test was performed. Subsequently, two-way analysis of variance (ANOVA) for the factors group (EG vs. CG) and time (PRE intervention vs. POST intervention) was performed to examine the interaction effect of the characteristics on fitness and body composition components. If any significant interaction was observed, Tukey’s post hoc test was applied to detect differences between groups. For ANOVA results, effect sizes were calculated using partial eta squared (η2p) and classified according to Cohen via the following interpretation scale: <0.2 [small]; 0.2 to < 0.8 [moderate]; > 0.8 [large]. Complementarily, post-intervention changes within and between groups were calculated via Cohen’s d as effect size (ES). Threshold values for Cohen’ d ES statistics were: < 0.20 [trivial], 0.20 [small], 0.60, [moderate], 1.20 [large], 2.0 [very large], and 4.0 [extremely large], respectively [35] using Hopkins’ spreadsheets with the 90% interval confidence (90%CI). Following the end of the intervention, pre–post intervention differences in delta (Δ) were calculated for each variable, and the sample was classified into responders (Rs) and non-responders (NRs) using the two-technical error (TE) criterion, according to a previously established equation [16]. NRs were identified and defined as individuals who were unable to demonstrate an increase or decrease (in favour of beneficial changes) in body composition and fitness variables that was greater than twice the TE away from zero. For the current study, two replicates of all variables analysed were used to calculate TE. A change beyond twice the TE was representative of a high probability (i.e., 12 to 1 odds) that the observed response was a true physiological adaptation beyond what might be expected as a result of technical and/or biological variability [14]. In addition, Fisher’s exact test was used to make comparisons between groups of subjects who were at 2 × TE calculated at each outcome (NRs) or more than twice the TE (Rs) [14]. The TEs were as follows: [SJ 2.16 (cm) × 2; CMJ 2.36 (cm) × 2; TSAT 0.48 (s) × 2; 5M 0.06 (m s^−1^) × 2; total kicks 3.93 kicks × 2; KDI 3. 60% × 2; 20MSR 0.44 (min) × 2; BM 0.94 (kg) × 2; %BM 2.24 (%) × 2; FM 1.34 (kg); MM 0.87 (kg) × 2]. Statistical significance was set at *p* < 0.05.

## 3. Results

### 3.1. Normality of the Results Analysed

The assumption of homoscedasticity of the results was verified in the totality of the results. Specifically, in general and specific physical fitness: SJ (F = 0.36; *p* = 0.77), CMJ (F = 2.23; *p* = 0.88), TSAT (F = 2.70; *p* = 0.64), 5M (F = 4.0; *p* = 0.17), total kicks (F = 0.39; *p* = 0.69), KDI (F = 1.21; *p* = 0.32), and 20MSR (F = 0.33; *p* = 0.80). In addition, in body composition, BM (F = 0.14; *p* = 0.71), FM% (F = 0.84; *p* = 0.77), FM (F = 0.64; *p* = 0.80), and MM (F = 0.05; *p* = 0.81).

### 3.2. Differences between Athletes in Both Groups at Baseline Assessments

No significant differences were documented in baseline assessments between groups in body composition characteristics: BM (*t* = 0.18; *p* = 0.85; ES = 0.09), FM% (*t* = −0.48; *p* = 0.63; ES = −0.24), FM (*t* = −0.72; *p* = 0.47; ES = −0.36), MM (t = 0.06; *p* = 0.94; ES = 0.33). Similarly, in the physical fitness components: SJ (*t* = 0.80; *p* = 0.41; ES = 0.41), CMJ (*t* = 0.29; *p* = 0.77; ES = 0.14), TSAT (*t* = 0.24; *p* = 0.89; ES = 0. 12), total kicks (*t* = 0.26; *p* = 0.79; ES = 0.13), KDI (*t* = −1.54; *p* = 0.14; ES = −0.77), 20MSR (*t* = 0.10; *p* = 0.91; ES = 0.05) with the exception of 5M (*t* = 2.38; *p* = 0.03; ES = 1.19).

### 3.3. Interaction between the Factors Analysed

Table 2 presents the summary of the interactions between the factors analysed for general and specific physical fitness and body composition.

Regarding the results of the two-factor ANOVA analysis: group (EG vs. CG) and time (PRE intervention and POST intervention), no significant differences were reported for the time factor, group factor, and interaction between the factors group by time. Specifically, general physical fitness presented the following results for SJ in the group factor (F = 0.78; *p* = 0.40; η^2^_p_ = 0.02) and in the time factor (F = 0.00; *p* = 0.93; η^2^_p_ = 0. 00), in CMJ for the group factor (F = 0.86; *p* = 0.36; η^2^_p_ = 0.02) and time factor (F= 1.68; *p* = 0.20; η^2^_p_ = 0.05), in 5M for the group factor (F = 1.05; *p* = 0.31; η^2^_p_ = 0.03) and time factor (F = 2.37; *p* = 0.01; η^2^_p_ = 0.07). In turn, specific fitness also exhibited no significant differences in TSAT for the group factor (F = 0.34; *p* = 0.56; η^2^_p_ = 0.00) and time factor (F = 10.3; *p* = 0.00; η^2^_p_ = 0.26), in total kicks for the group factor (F = 0.08; *p* = 0.77; η^2^_p_ = 0. 00) and time factor (F = 1.47; *p* = 0.23; η^2^_p_ = 0.05), in KDI for the group factor (F = 4.06; *p* = 0.05; η^2^_p_ = 0.10) and time factor (F = 6.74; *p* = 0.01; η^2^_p_ = 0.17), and in 20MSR for the group factor (F = 0.08; *p* = 0.76; η^2^_p_ = 0.00) and time factor (F = 1.80; *p* = 0.19; η^2^_p_ = 0.06).

There were also no significant differences reported in body composition, specifically in BM for the group factor (F = 0.05; *p* = 0.81; η^2^_p_ = 0.00) and time factor (F = 0.10; *p* = 0.74; η^2^_p_ = 0.00), in FM% for the group factor (F = 0.92; *p* = 0.35; η^2^_p_ = 0. 03) and time factor (F= 0.00; *p* = 0.95; η^2^_p_ = 0.00), in BF for group factor (F = 1.19; *p* = 0.28; η^2^_p_ = 0.04) and time (F = 0.14; *p* = 0.70; η^2^_p_ = 0.00), in MM for group factor (F = 0.06; *p* = 0.80; = η^2^_p_ = 0.02) and time factor (F = 0.01; *p* = 0.90; η^2^_p_ = 0.00). Similarly, no significant interaction was documented for the factors group by time.

### 3.4. Magnitude of Change Based on Inference

Following the intervention period, increases in TSAT physical performance were reported in both groups, moderately decreasing test performance times in the EG (−8.6%; ES = −1.07) and in the CG (−12.7%; ES = −0.87), with a trivial difference of 0.8% in favour of the EG (ES = 0.06). In the FSKT_MULT_ test, an increase for total kicks was documented in the EG (4.4%; ES = 0.58) and in the CG (3.7%; ES = 0.36), with a trivial difference of 0.8% in favour of the EG (ES = 0.09). In addition, a moderate decrease in KDI in the EG (−32.5%; ES = −0.80) and a low decrease in the CG (−37.7%; ES = −0.48), with a trivial difference of −11.8% in favour of the CG (ES = −0.17). In relation to 20MSR performance, a moderate increase in time (min executed) was reported in EG (12.9%; ES = 1.07) and low in CG (9.2%; ES = 0.24) with a trivial difference of 2% in favour of the EG (ES = −0.06).

On the other hand, decreases in jump height were documented in SJ with a trivial decrease in the CG (−1.7%; ES = −0.07). In CMJ, a moderate decrease in jump height was documented in the EG (−10.4%; ES = −0.83) vs. a small decrease in the CG (−9.6%; ES = −0.36) with a low difference of 7.6% in favour of the EG (ES = 0.34). In turn, a moderate decrease in 5M performance was documented in the CG (7.5%; ES = 1.11) and moderate in the EG (−3.8%; ES = −0.52) with a trivial difference of 8.5% (ES = 1.27) between groups.

With respect to body composition characteristics, a small increase in FM% was documented in the EG (19.5%; ES = 0.45). Trivial decreases in MM were documented in the CG (−0.8%; ES = −0.02) and in the EG (−3.1%; ES = −0.17) with differences of 1.7% (ES = 0.06) in favour of the CG.

### 3.5. Inter-Individual Variability in Response to the Intervention

**Figure 3:**
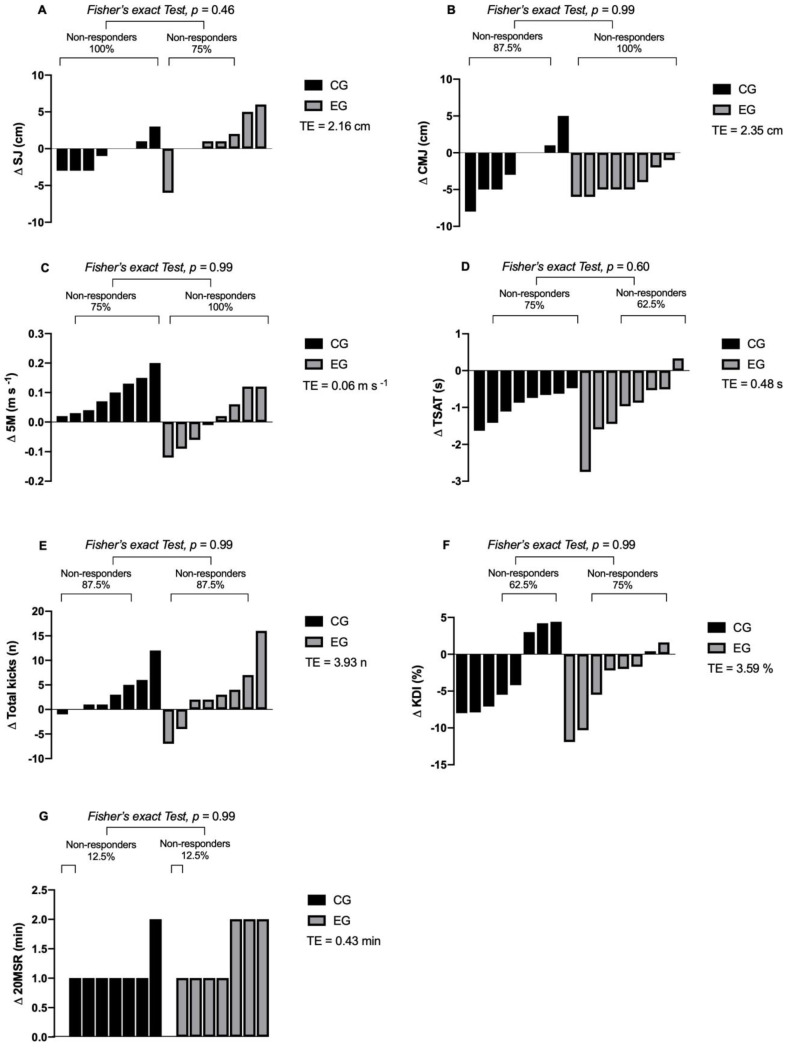
Inter-individual variability of the response of HIIT intervention and traditional training on the physical fitness of taekwondo athletes. Means: *p*: *p* value; TE: technical error; Δ: delta or change post-pre. Symbolises: (**A**) squat jump; (**B**) countermovement jump; (**C**) 5M linear sprint in 5 metre; **D**: TSAT: taekwondo specific agility test; (**E**) total kicks; (**F**) KDI: kick decreased index; (**G**) 20MSR: 20-metre shuttle run.

Regarding the inter-individual response of both groups, responders were documented for SJ (EG: *n* = 2, 25%), CMJ (CG: *n* = 1, 12.5%), TSAT (EG: *n* = 3, 37.5%; CG: 2, 25%), KDI (EG: *n* = 2, 25%; CG: *n* = 3, 37.5%), Total kicks (EG: *n* = 1, 12.5%; CG: *n* = 1, 12.5%), 20MSR (EG: *n* = 7, 87.5%; CG: *n* = 7, 87.5%), and BM (EG: *n* = 4, 50%; CG: *n* = 3, 37.5%), FM (EG: *n* = 1, 12.5%; CG: *n* = 1, 12.5%), FM% (EG: *n* = 1, 12.5%; CG: *n* = 1, 12.5%), and MM (CG: *n* = 1, 12.5%) outcomes, which are shown in detail in Table 3 and Figure 3.

**Figure 4:**
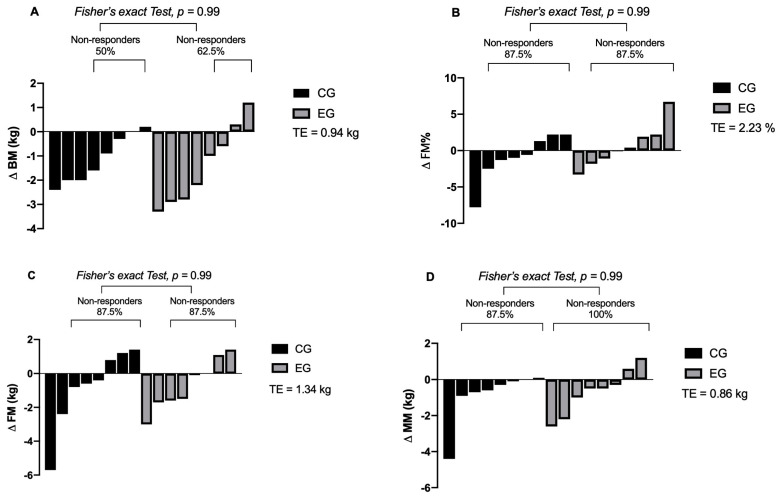
Inter-individual variability of the response of HIIT intervention and traditional training on body composition in taekwondo athletes. Means: *p*: *p*-value; TE: technical error; percentage change expressed as percentage delta. Symbolises: (**A**) BM: body mass, (**B**) FM%: fat mass percentage, (**C**) FM: fat mass; (**D**) MM: muscle mass.

On the other hand, non-responders were reported for SJ (CG: *n* = 6, 75%; EG: *n* = 8, 100%), CMJ (EG: *n* = 8, 100%; CG: *n* = 7, 87.5%), TSAT (EG: *n* = 2, 62.5%; CG: *n* = 6, 75%), 5M (EG: *n* = 8, 100%; CG: *n* = 6, 75%), total kicks (EG: *n* = 7, 87%; CG: *n* = 7, 87%), KDI (EG: *n* = 6, 75%; CG: *n* = 5, 62.5%), 20MSR (EG: *n* = 1, 12.5%; CG: *n* = 1, 12.5%), BM (EG: *n* = 4, 50%; CG *n* = 5, 62.5%), FM (EG: *n* = 7, 87.5%; CG: *n* = 7, 87.5%), FM% (EG: *n* = 7, 87.5%; CG: *n* = 7, 87.5%), and MM (EG: *n* = 8, 100%; CG: *n* = 7, 87.5%) outcomes, which are shown in detail in Table 3 and Figure 4.

## 4. Discussion

The main purpose of the present study was to compare the effects of a technique-specific HIIT protocol vs. traditional taekwondo training on general and specific physical fitness and body composition in taekwondo athletes and specifically, to analyse the inter-individual response of the athletes. Among the main findings, no significant differences (*p* > 0.05) were reported in the components analysed between the groups. Meanwhile, in the inter-individual response, responders and non-responders were observed in the EG and CG, although without statistically significant differences between the groups.

### 4.1. Changes in Jumping Performance

In relation to the jump ability results obtained through performance in SJ and CMJ, no significant interactions were documented between the groups by time factors. Although in SJ, EG improved by 2.6% (ES = 0.20), where 25% of athletes were classified as responders. However, these results contrast with the current evidence. In fact, significant increases in jump height in these components of physical fitness are observed after the application of HIIT protocols in taekwondo based on technique-specific movements [12] and also based on repeated sprinting [9,11]. In the study by Monks et al. [9], the authors applied two to three additional sessions per week using repeated 60 s sprints with two-minute breaks after four weeks of training. Similarly, in the study by Ouergui et al. [12], the technique-specific HIIT group performed two additional sessions of three sets of ten repetitions of six s with ten s of passive pause with three-min rests during four weeks of training. HIIT, through the application of sustained high-intensity efforts (>80–90% at VO2 max), stimulates the recruitment of fast fibres and the neuromuscular system, increasing performance in these components [36,37]. Therefore, it is likely that the volume of the HIIT protocol developed was insufficient to achieve significant results in these abilities.

#### 4.1.1. TSAT and 5M

On the other hand, in relation to the results of specific agility assessed by the TSAT test, no significant interactions (*p* > 0.05) between the groups by time factors were reported. However, the EG improved by −8.6% (ES = −1.07) and the CG improved by −12.7% (ES = −0.87). In addition, both groups reported responders. This is important to analyse as, similarly, Ouergui et al. [12] recently reported significant increases in this skill following the application of technique-specific HIIT vs. traditional training for six weeks. In other agility tests—specifically change of direction as assessed by the T-test—significant increases in performance are also reported four weeks following the application of repeated four-week sprint-based HIIT protocols in taekwondo athletes [9]. Consequently, agility is considered a relevant complex skill in combat sports and a prerequisite in taekwondo for success in high performance, enabling technical–tactical movements in multiplanar directions [23,38]. This skill is characterised by the ability to maintain and control body position correctly while rapidly changing position through a series of movements [23]. In turn, this ability is dependent on other factors including balance, dynamic muscle actions, and cognitive factors [23]. Therefore, the trend towards a reduction in TSAT performance time exhibited by the taekwondo athletes in our study is auspicious, due to the specificity of the stimulus applied, which could influence the response observed for both groups [13].

#### 4.1.2. FSKT_MULT_

Continuing with the analysis of the components of the specific physical fitness, no significant interactions were reported between group-by-time factors. However, in the KDI score, the EG improved by −32.5% (ES = −0.80) and the CG improved by −37.7% (ES = −0.48). Similarly, in total kicks, the EG improved by 4.4% (ES = 0.58) and the CG improved by 3.7% (ES = 0.36). These results are partially similar to those reported by Aravena et al. [11], who after four weeks of applying an additional technique-specific HIIT protocol to training (3 blocks of 6 repetitions of 10 s of all-out effort with 10 s pause between sets and 1 min of passive rest interval between blocks) reported significant changes in this test. In this sense, the addition of HIIT sessions to the usual training reported by Aravena et al. [11] could explain these differences. In taekwondo, anaerobic fitness is an important characteristic to develop because punches and kicks are applied with high-intensity movement [39]. In addition, a decrease in KDI represents an increase in the ability to sustain high-intensity efforts [39]. In addition, an increase in total kicks is indicative of improved physical performance in this skill [39].

#### 4.1.3. 20MSR

Regarding the 20MSR results, no significant interactions between group-by-time factors were reported. However, performance increases were reported in the EG by 12.9% (ES = 1.07) and in the CG by 9.2% (ES = 0.24). In addition, responders were observed in the majority of athletes in both groups. These results partially contrast with the study by Ouergui et al. [12], who report significant increases in the performance of this test after four weeks of application of a technique-specific HIIT protocol based on repeated sprinting, although without significant interactions with the control group, requiring further research to corroborate the effectiveness of this capacity.

Cardiorespiratory fitness is an important component of physical fitness for performance in combat sports due to the predominant contribution of the oxidative system [8,9,40,41]. Furthermore, according to Franchini et al. [8], this component is important for maintaining the volume and intensity of attacks during bouts, allowing a rapid resynthesis of creatine phosphate during the short pauses between the high-intensity actions performed, which allows a faster recovery between successive bouts. On the other hand, HIIT is characterised by the use of high-intensity stimuli with the purpose of spending most of the time of the protocol at high VO2max, this being a powerful stimulus to develop central adaptations (oxygen transport) and peripheral adaptations (oxygen utilisation) [18,42].

#### 4.1.4. Changes in Body Composition

In relation to body composition, no significant group-by-time factor interactions were reported, although inter-individual differences were found in both groups independently. The low effectiveness of the HIIT intervention on body composition is consistent with previous reports in combat sports. Specifically, in taekwondo athletes, Monks et al. [9] reported no significant differences in FM% after comparing a repeated sprint-based HIIT protocol (3 sets of 60 s at 85–100% HRmax with 120 s) with a continuous moderate-intensity training (5 km at 85% of HRmax). Similarly, using technique-specific HIIT protocols in judo after four weeks [43] and in boxing after one month of intervention [44], no significant differences were found with the control groups. In this regard, it is important to consider that these studies did not establish a nutritional intervention, which is a limitation that is also present in our study. However, these results are supported by evidence from high-level combat sports athletes, indicating that no body composition adaptations are generated in short periods of intervention (4–12 weeks) [8]. In this regard, Keating et al. [45] through a systematic review with meta-analysis have determined that HIIT is no more efficient than moderate training in reducing FM, FM%, and visceral fat. On the other hand, in overweight and obese populations, HIIT seems to be more efficient in reducing FM% and FM [46]. In this context, evidence suggests that in addition to the energy expenditure of physical exercise, it is important to maintain a negative energy balance, i.e., a caloric intake that does not exceed total energy expenditure, in order to modulate body composition [45,46,47]. This is still a controversial issue [48].

### 4.2. Limitations

Possible limitations of the study include the following: (i) the lack of control of intensity by physiological measures. In this sense, although the HIIT all-out format is accepted in the scientific literature, taekwondo athletes may have underestimated the intensity of the work performed; (ii) the lack of control of dietary habits that could have influenced the reported changes in body composition; (iii) the lack of progression of the training load applied in HIIT; (iv) the evaluation by bioimpedance, which could have overestimated the body composition; and (v) the small number of athletes analysed. In addition, potential biases that could have influenced the results of this study include the following: (i) the variability of the response according to the sex of the athletes [49,50,51]; (ii) the absence of analysis and subsequent distribution of the athletes according to their biological age [52,53]; (iii) the variation of physical performance according to the time of day [54]; and (vi) the possible variability of the body composition due to the use of bioimpedance [55].

To address the limitations and biases of this study, future research could (i) use heart monitors or other physiological monitoring methods to verify compliance with training intensity; (ii) monitor dietary habits; (iii) apply more physiological stress by increasing the training load (e.g., increased number of sets, duration of bouts) [56]; (iv) consider studying a larger number of athletes; (v) perform independent statistical analyses by gender; (vi) assess biological age in addition to chronological age; (vii) homogenise the assessment and application of training protocols by time of day; and (viii) use the four-compartment model to assess body composition including air plethysmography, Dual X-ray Absorptiometry, and dilution techniques [57,58].

### 4.3. Highlights

In accordance with the above, it is relevant to point out that to the best of our knowledge, this is the first randomised controlled trial to apply a technique-specific HIIT protocol that adhered to the regular taekwondo training session, using the total duration of the bout and analysing the inter-individual response on general and specific physical fitness and body composition in athletes. Although no significant differences were found, this study reveals the need for further research in this area. In fact, most of the studies with HIIT in combat sports [8], which report significant differences in the aforementioned variables, complement the training with an extra HIIT session [9,10,11,12].

Although requiring further study, HIIT protocols based on taekwondo-specific technical movements and using the temporal structure of combat could be an alternative to incorporate as part of the training session. Since, in a short period of time, coaches could maintain or improve fitness components, which would help in pre- and inter-competition training (i.e., shock microcycle), due to the limited time available for athletes to cope with the demands of this period.

Although, in particular, this trial addresses three questions that may be important to develop in future research: (i) the potential efficacy of using technique-specific work stimuli during HIIT; (ii) the use of the appropriate time structure of the bout as a work interval during HIIT; (iii) the efficacy of the application of HIIT during the training session.

Moreover, coaches could use inter-individual response analysis as a practical monitoring tool to track each athlete’s progress against the training programme in order to understand and document individual response, which would assist in modifications or remediation to improve performance. Therefore, researchers are encouraged to conduct further studies on this research topic.

## 5. Conclusions

A four-week HIIT protocol based on taekwondo-specific technical movements does not report significant differences in general and specific physical fitness and body composition compared to traditional training in taekwondo athletes. However, there was a higher percentage of responders in the EG compared to the CG, which is promising for future research.

## Figures and Tables

**Table 1:** Description of the load programming of the training protocols carried out.

	EG (*n* = 8)	CG (*n* = 8)
1st week	3 rounds of 4 repetitions of 4 s of work: 28 s rest/1 min recovery	Continuous roundhouse kick with partner with use of paddles for speed
2nd week	3 rounds of 5 repetitions of 4 s of work: 24 s rest/1 min recovery	Continuous bandal-tchagi kicks with partner with use of paddle for speed
3rd week	3 rounds of 5 repetitions of 4 s of work: 20 s rest/1 min recovery	Simulated combat with technical specifications
4th week	3 rounds of 6 repetitions of 4 s of work: 16 s rest/1 min recovery	Simulated combat with technical specifications

Simbolises: EG: experimental group; CG: control group.

**Table 2:** Pre- and post-intervention differences between HIIT vs. traditional training protocols on physical fitness and body composition in taekwondo athletes (*n* = 16).

	PRE	POST	PRE	POST	F	*p*	η^2^_p_
**Physical Fitness Components**							
SJ (cm)	30 ± 6.8	29.2 ± 5.2	27.2 ± 6.2	28.3 ± 5.5	0.19	0.66	0.00
CMJ (cm)	33.3 ± 7.4	31.5 ± 6.4	32.3 ± 6.2	28.1 ± 6.44	0.25	0.61	0.09
5M (m s^−1^)	1.14 ± 0.07	1.24 ± 0.09	1.22 ± 0.05	1.23 ± 0.12	1.91	1.91	0.64
TSAT (s)	7.95 ±1.11	5.0 ± 0.74	7.82 ± 1.04	6.78 ± 0.42	1.91	0.66	0.01
Total kicks (n)	95.3 ± 7.2	98.7 ± 7.3	96.3 ± 7.8	99.2 ± 6.5	0.09	0.92	0.00
KDI (%)	7.9 ± 3.9	5.2 ± 3.2	11.1 ± 4.3	7.1 ± 2.4	1.80	0.60	0.00
20MSR (min)	7.8 ± 2.6	8.8 ± 2.5	8.0 ± 2.1	9.2 ± 2.0	0.02	0.88	0.01
**Anthropometric and Body Composition Characteristics**							
BM (kg)	61.8 ± 11.5	60.6 ± 11.2	62.8 ± 11.1	61.43 ± 9.9	0.001	0.97	0.00
FM (kg)	10.6 ± 5.6	9.8 ± 5.5	12.7 ± 5.6	12 ± 5.1	0.20	0.92	0.01
FM (%)	17.0 ± 7.9	16.1± 8.2	19.3 ± 10.4	19.9 ± 8.6	0.23	0.63	0.00
MM (kg)	28.5 ± 6.2	28.6 ± 6.7	28.2 ± 7.3	27.6 ± 6.4	0.36	0.85	0.00

Data are presented as mean ± standard deviation. CG: control group EG: experimental group; PRE: before the intervention; POST: post-intervention. F: f-value interaction between factors time by group; *p*: *p* value; η^2^_p_: partial square eta; SJ: squat jump; CMJ: countermovement jump; TSAT: taekwondo specific agility test; KDI: kick decreased index; 20MSR: 20-metre shuttle run; BM: body mass; FM: fat mass; FM%: fat mass percentage; MM: muscle mass.

**Table 3:** Differences and rates of responders and non-responders to HIIT and traditional training interventions.

	EG (*n* = 8)			CG (*n* = 8)			EG vs. CG	
	Δ % (90%CI)	ES (90%CI)	Rs, *n* (%)	Δ % (90%CI)	ES (90%CI)	Rs, *n* (%)	Δ % (90%CI)	ES (90%CI)
**Physical Fitness Components**								
SJ (cm)	2.6 (0.1 to 5.3)	0.20 (0.01 to 0.40)	2 (25)	−1.7 (−8.8 to 5.9)	−0.07 (−0.35 to 0.22)	0 (0)	−5.2 (−15.7 to 6.6)	−0.20 (−0.64 to 0.24)
CMJ (cm)	−10.4 (−15.5 to −5.0)	−0.83 (−1.27 to −0.39)	0 (0)	−9.6 (−19.7 to 1.7)	−0.36 (−0.78 to 0.06)	1 (12.5)	11 (−0.7 to 24.2)	0.45 (−0.03 to 0.93)
TSAT (s)	−8.6 (−16.5 to 0.00)	−1.07 (−2.14 to 0.00)	3 (37.5)	−12.7 (−16 to −9.2)	−0.87 (−1.12 to −0.61)	2 (25)	0.8 (−6.9 to 9.1)	0.06 (−0.48 to 0.60)
5M (m s^−1^)	−3.8 (−9.0 to 1.8)	−0.52 (−1.28 to 0.24)	0 (0)	7.5 (1.8 to 13.5)	1.11 (0.27 to 1.94)	2 (25)	−8.5 (−13.6 to −3.0)	−1.27 (−2.10 to −0.44)
Total kicks (*n*)	4.4 (−3.6 to 13.0)	0.58 (−0.49 to 1.64)	1 (12.5)	3.7 (−1.6 to 9.4)	0.36 (−0.16 to 0.88)	1 (12.5)	0.8 (−4.5 to 6.3)	0.09 (−0.53 to 0.72)
KDI (%)	−32.5 (−56.4 to 4.5)	−0.80 (−1.69 to 0.09)	2 (25)	−37.7 (−79.8 to 92.1)	−0.48 (−1.63 to 0.67)	3 (37.5)	−11.8 (−66.4 to 131.8)	−0.17 (−1.48 to 1.14)
20MSR (min)	12.9 (4.0 to 22.6)	1.07 (0.34 to 1.79)	7 (87.5)	9.2 (3.4 to 15.3)	0.24 (0.09 to 0.39)	7 (87.5)	−2.0 (−10.9 to 7.7)	−0.06 (−0.34 to 0.22)
**Body Composition Characteristics**								
BM (kg)	−2.1 (−4.7 a 0.4)	−0.12 (−0.26 to 0.02)	4 (50)	−1.7 (−3.4 to 0.1)	0.05 (−0.11 to 0.00)	3 (37.5)	0.3 (−1.6 to 2.4)	0.01 (−0.11 to 0.13)
FM (kg)	2.2 (−18.8 to 17.7)	0.05 (−0.48 to 0.38)	1 (12.5)	3.6 (−16.1 to 10.8)	0.04 (−0.22 to 0.13)	1 (12.5)	6.9 (−11.5 to 29.1)	0.11 (−0.20 to 0.42)
FM (%)	19.5 (−8.8 to 56.5)	0.45 (−0.23 to 1.12)	1 (12.5)	−2.0 (−13.8 to 11.5)	−0.03 (−0.20 to 0.15)	1 (12.5)	−10.0 (−24.8 to 7.8)	−0.17 (−0.46 to 0.12)
MM (kg)	−3.1 (−6.6 to 0.5)	−0.17 (−0.37 to 0.03)	0 (0)	−0.8 (−3.9 to 2.3)	−0.02 (−0.11 to 0.06)	1 (12.5)	0.8 (−2.5 to 4.2)	0.03 (−0.09 to 0.16)

Data are presented as mean ± standard deviation. EG: experimental group; CG: control group. Rs: responders; Δ %: change expressed as percentage delta; ES: effect size; 90%CI: 90% confidence interval. SJ: squat jump; CMJ: countermovement jump; TSAT: taekwondo specific agility test; total kicks; KDI: kick decreased index; 20MSR: 20-metre shuttle run; BM: body mass; FM: fat mass; FM%: percentage fat mass; MM: muscle mass.

## Data Availability

The data presented in this study are available on request from the corresponding author.
